# Optimizing brushless direct current motor design: An application of the multi-objective generalized normal distribution optimization

**DOI:** 10.1016/j.heliyon.2024.e26369

**Published:** 2024-02-15

**Authors:** Sundaram B. Pandya, Pradeep Jangir, Miroslav Mahdal, Kanak Kalita, Jasgurpreet Singh Chohan, Laith Abualigah

**Affiliations:** a Department of Electrical Engineering, Shri K.J. Polytechnic, Bharuch 392 001, India; b Department of Biosciences, Saveetha School of Engineering. Saveetha Institute of Medical and Technical Sciences, Chennai 602105, India; c Department of Control Systems and Instrumentation, Faculty of Mechanical Engineering, VSB-Technical University of Ostrava, 17. Listopadu 2172/15, 70800 Ostrava, Czech Republic; d Department of Mechanical Engineering, Vel Tech Rangarajan Dr. Sagunthala R&D Institute of Science and Technology, Avadi 600 062, India; e Department of Mechanical Engineering and University Centre for Research & Development, Chandigarh University, Mohali, 140413, India; f Computer Science Department, Prince Hussein Bin Abdullah Faculty for Information Technology, Al al-Bayt University, Mafraq 25113, Jordan; g Hourani Center for Applied Scientific Research, Al-Ahliyya Amman University, Amman 19328, Jordan; h MEU Research Unit, Middle East University, Amman, Jordan; i School of Computer Sciences, Universiti Sains Malaysia, Pulau Pinang 11800, Malaysia; j Department of Electrical and Computer Engineering, Lebanese American University, Byblos 13‑5053, Lebanon; k School of Engineering and Technology, Sunway University Malaysia, 27500 Petaling Jaya, Malaysia; l Artificial Intelligence and Sensing Technologies (AIST) Research Center, University of Tabuk, 71491 Tabuk, Saudi Arabia; m Applied Science Research Center, Applied Science Private University, Amman 11931, Jordan

**Keywords:** BLDC motor, Electromagnetics, Metaheuristic, Non-dominated sorting generalized normal distribution optimization

## Abstract

In this study, we tackle the challenge of optimizing the design of a Brushless Direct Current (BLDC) motor. Utilizing an established analytical model, we introduced the Multi-Objective Generalized Normal Distribution Optimization (MOGNDO) method, a biomimetic approach based on Pareto optimality, dominance, and external archiving. We initially tested MOGNDO on standard multi-objective benchmark functions, where it showed strong performance. When applied to the BLDC motor design with the objectives of either maximizing operational efficiency or minimizing motor mass, the MOGNDO algorithm consistently outperformed other techniques like Ant Lion Optimizer (ALO), Ion Motion Optimization (IMO), and Sine Cosine Algorithm (SCA). Specifically, MOGNDO yielded the most optimal values across efficiency and mass metrics, providing practical solutions for real-world BLDC motor design. The MOGNDO source code is available at: https://github.com/kanak02/MOGNDO.

## 1 Introduction

Brushless Direct Current (BLDC) motor has garnered significant attention for its unique feature of electronic commutation, making it indispensable in a myriad of applications ranging from automotive and consumer electronics to robotics and aerospace [1,2]. The escalating demand for energy efficiency, high power density, and minimal maintenance has catalyzed the adoption of BLDC motors, superseding their brushed counterparts [3,4]. However, the design intricacies of BLDC motors present a formidable challenge [5,6]. Unlike conventional DC motors, where brushes and commutators have been replaced by electronic controllers, the BLDC motor employs high-grade permanent magnets in its stationary armature to obviate the need for energizing the rotating armature [7,8].

Designing a BLDC motor is a multi-faceted task, influenced by a plethora of parameters such as size, weight, cost, power output, and efficiency [9,10]. These parameters often pose a trade-off dilemma; for instance, augmenting power output may inadvertently increase the motor's size and weight, which is undesirable in applications requiring compactness, such as drones [11,12]. Similarly, elevating efficiency could inflate costs, rendering the solution economically impractical. The advantages of BLDC motors are manifold, including superior torque-to-watt and torque-to-weight ratios, reduced acoustic noise, and exceptional reliability. To ascertain the rotor's precise position, rotary encoders or Hall-effect sensors are commonly employed [13]. Although the initial investment in a BLDC motor may exceed that of its brushed counterpart, the long-term operational efficiency justifies the capital expenditure.

In the realm of electromagnetic design, analytical models often supersede Finite Element Analysis (FEA) for their computational efficiency and adaptability [14]. However, these models are not without limitations, particularly in addressing complex design challenges. This has led to the burgeoning adoption of metaheuristic algorithms for electromagnetic optimization, especially in scenarios characterized by multiple local optima [15,16]. In addition to these, several researchers have delved into diverse methodologies and techniques to optimize and control BLDC motors. Jadhao et al. [17] explored the use of Artificial Neural Networks (ANN) for controlling PMBLDC motors in electric vehicles using a zeta converter, emphasizing battery power optimization and sensorless speed control. Ahmed et al. [18] presented a detailed study of PMBLDC motor mechanisms, focusing on the dynamic load characteristics and optimization for 126 kV vacuum circuit breaker applications.

Recent literature has explored various metaheuristic algorithms for BLDC motor design optimization, including the Bat Algorithm (BA) [19], Multi-Objective Krill Herd (MOKH), and Non-Dominated Sorting Genetic Algorithm-Version II (NSGA-II). However, these traditional methods exhibit limitations in handling conflicting objectives and achieving global optima [[20], [21], [22]]. Yan et al. [23] introduced a self-adaptive JAYA (SAJAYA) algorithm to optimize BLDC motor design parameters, enhancing efficiency and preventing premature convergence. Vinida and Chacko [24] implemented a sensorless BLDC motor drive using H-infinity control optimized by particle swarm optimization, demonstrating robust performance against disturbances. Karthika et al. [25] proposed an Arithmetic Optimization Algorithm (AOA) to mitigate torque ripple in BLDC drives, enhancing their application in domestic and industrial settings. Knypiński et al. [26] explored optimal designs for BLDC motors for electromobility propulsion, comparing exterior and interior rotor machines using the Taguchi method. Rajesh et al. [27] introduced an improved jellyfish Search (ImpJS) technique for torque ripple minimization in BLDC motors controlled by a CUK converter. Niu et al. [28] developed a hybrid teaching-learning-based optimization algorithm for optimal electromagnetic design, including BLDC motor design and electromagnetic actuator construction.

Further, Rajesh et al. [29] proposed an enhanced DC-DC converter with hybrid control for torque ripple minimization in BLDC motors, integrating the Enhanced Artificial Transgender Longicorn Algorithm (EATLA) and Recurrent Neural Network (RNN). Saravanan et al. [30] presented a hybrid algorithm combining Particle Swarm Optimization (PSO) with Takagi Sugeno adaptive fuzzy interference system and gravitational search algorithms for BLDC motor speed control. Anish and Nisha [31] explored the use of ZSI with the Functional Order Proportional Integral Derivative (FOPID) controller optimized by the Emperor Penguin optimization (EPO) algorithm for BLDC motor control in solar-powered applications. Pakdeeto et al. [32] proposed an adaptive Tabu Search algorithm for system identification and PID speed controller design for BLDC motors, emphasizing real device limitations. Lins and Krishnakumar [33] presented a comprehensive approach to BLDC motor control in solar-powered electric vehicles, integrating Zeta converter, optimization algorithms, and FPGA-based control. Lastly, Kumar et al. [34] developed a novel method combining space vector pulse width modulation (SVPWM) with direct torque control (DTC) using an optimal PI controller for BLDC motor control, achieving reduced torque ripple and improved speed regulation.

This paper introduces the Multi-Objective Grey Wolf Non-Dominated Optimization (MOGNDO) technique, a stochastic evolutionary computation method, to address these challenges. The MOGNDO algorithm leverages non-dominated sorting and crowding distance methodologies to enhance solution accuracy and convergence speed [35]. The primary contributions of this research are:

• Application of the MOGNDO algorithm to optimize BLDC motor design, evaluated against established multi-objective algorithms.
• Comparative performance analysis based on seven DTLZ test functions and real-world BLDC motor design challenges.

The remainder of this paper is structured as follows: Section 2 articulates the problem formulation; Section 3 elucidates the fundamental principles of MOGNDO in the context of BLDC motor design; Section 4 presents simulation results and comparative analyses; and Section 5 concludes the study.

## 2 Overview and mathematical representations of BLDC motor

Brushless Direct Current (BLDC) motors have become increasingly vital in a range of applications due to their superior characteristics such as high efficiency, longer operational life, compactness, and high reliability.

BLDC motors operate on the principle of electronically controlled commutation system as opposed to the traditional mechanical commutation used in brushed motors. A typical BLDC motor consists of a rotor with permanent magnets and a stator with windings. The electronic controller performs the power switching in the windings, creating a rotating electric field that drives the rotor.

The performance of a BLDC motor is influenced by various parameters:

Power Output: This is the mechanical power that the motor delivers at the output shaft. It is a critical parameter for the motor's operation and is influenced by factors like the magnetic field strength, the number of windings, and the current.

Efficiency: This is the ratio of the output power to the input power. High efficiency is crucial as it means less energy is wasted, making the motor more sustainable and cost-effective.

Size and Weight: The size and weight of the motor are critical for applications where space and weight constraints are significant, such as in drones or electric vehicles. Optimizing the design can help reduce the size and weight without compromising the motor's performance.

Cost: The cost of the motor involves the expense of materials, manufacturing, and maintenance. Minimizing cost while maintaining high performance is a critical aspect of motor design.

Optimizing these parameters poses a significant challenge as they are often interrelated and improvement in one aspect can lead to the compromise of another. For instance, increasing power output might necessitate a larger size or higher cost, which might not be desirable. Therefore, optimizing a BLDC motor design involves striking a delicate balance among these conflicting objectives. As previously noted, BLDC motors are the preferred choice over traditional brushed DC motors due to their brushless operation, enhanced durability, superior torque production, and higher power handling capability. Their compact structure makes them highly suitable for applications in the transportation and electronics industries. The design process of Brushless Direct Current (BLDC) motors represents a widely acknowledged and frequently encountered optimization design dilemma. The complexity of the BLDC motor optimization problem is evident in the sheer number of non-linearities it embodies, comprising 78 non-linear equations that include five structural design dimensions. Prevailing strategies to tackle this issue often interpret the problem as an electrical benchmark, contemplating both singular and multiple objective scenarios. Notably, in comparison to the multi-objective problem, the single-objective issue only incorporates five constraints, significantly reducing its complexity.

The main goal is to maximise efficiency by optimizing the following design parameters: maximum current ($I_{\max}$), total mass ($M_{tot}$), temperature ($T_{a}$), inner diameter ($D_{\mathrm{int}}$), outer diameter ($D_{ext}$). The total mass ($M_{tot}$) is regarded as an objective function for minimization in an optimization problem for a multi-objective BLDC motor. For the motor to be seamlessly integrated into a wheel rim without causing demagnetization of the magnet, certain parameters need to be adhered to. The outer diameter should be restricted to less than 340 mm, while the maximum current ($I_{\max}$) should be equivalent to 125 A, and the total mass $(M_{tot}$) should remain below 15 kg. Moreover, for mechanical considerations, the inner diameter ($D_{\mathrm{int}}$) is required to exceed 76 mm, which is equivalent to five times the full load current. This necessity stems from the various mechanical constraints intrinsic to the motor design. Therefore, during the BLDC motor design process, optimization is required for five design parameters: $B_{e}$, $D_{s}$, $B_{d}$, $B_{cs}$, and ζ. Meanwhile, six inequality constraints need to be maintained. These include the ratio of the rotor length on one stator part (rrs), motor magnetic length (Lm), air-gap (e), input voltage (Vdc), number of pole pairs (P), and average magnetic induction in the yoke of the rotor (Bcr). These criteria ensure optimal functioning of the motor, providing a balance between performance and physical constraints.

The objective function used to guide the optimization process is presented in Eq. (1). This equation serves as the guiding mathematical principle to assist in navigating the complex interplay between these various parameters and constraints during the design and optimization process. This intricate task is pivotal in creating an efficient and fit-for-purpose BLDC motor.

(1) $$\begin{array}{c} \;\text{Maximize}\;\to f_{1}\left(\eta \right)\\ \;\text{Minimize}\;\to f_{2}\left(M_{tot}\right)\\ M_{tot}\leq 15\text{Kg},D_{ext}\leq 340\text{mm}\\ D_{\mathrm{int}}\geq 76\text{mm},I_{\max}\geq 125A\\ T_{a}\leq 120^{\circ }C,\text{discr}\;\left(D_{s},B_{d},B_{e},\zeta \right)\geq 0\\ 150\text{mm}\leq D_{s}\leq 330\text{mm},0.5T\leq B_{e}\leq 0.76T\\ 2A/{\text{mm}}^{2}\leq \zeta \leq 5A/{\text{mm}}^{2},0.9T\leq B_{d}\leq 1.8T\\ 0.6T\leq B_{cs}\leq 1.6T \end{array}$$

It can be seen from Eq. (1) that there are two goals: minimizing the overall mass (f2 ($M_{tot}$)) and maximizing the motor efficiency (f1(η)), with the restriction that $M_{tot}$ = 15 kg.

## 3 Generalized normal distribution optimization algorithm

### 3.1 Inspiration

The normal distribution theory, often called the Gaussian distribution, lays the groundwork for GNDO. It offers a powerful means to describe and analyze natural phenomena its probability density function may be written as Eq. (2):

(2) $$f\left(x\right)=\frac{1}{\sqrt{2\pi }\delta }\exp\left(-\frac{{\left(x-\mu \right)}^{2}}{2\delta^{2}}\right)$$

At its core, a normal distribution is the mathematical representation of a normal random variable, usually marked as ‘x'. The defining features of a normal distribution function are two parameters - the location parameter and the scale parameter, as described in Eq. (2). The location parameter symbolizes the mean value, while the scale parameter points to the standard deviation of random variables. Together, they delineate the dispersion and bias of the data points within a distribution. Understanding normal distribution proves to be particularly advantageous when examining population-based optimization strategies. Such strategies typically incorporate three distinct stages in the search procedures. The initial stage is characterized by a distributed pattern that includes all the nascent members of the population. Under the influence of carefully designed tactics of exploration and exploitation, each member initiates their quest towards the optimal solution, also known as the global optimum. The progression of this quest is portrayed through multiple normal distributions, accurately encapsulating the essence of the search process. To clarify, one could imagine each individual's position in this journey as a random variable adhering to a normal distribution. At the outset, a significant disparity exists between the ideal and the mean positions, reflecting a high standard deviation in the locations of all members. As the process advances to the second stage, this disparity begins to contract, with a parallel reduction in the standard deviation related to each individual's position. The culmination of the process is signaled by the third stage, where all individuals congregate around the global optimal solution. This stage witnesses the narrowest gap between the mean and the ideal positions, suggesting the lowest feasible standard deviation for each individual's location. It underscores the successful completion of the optimization process, with each member of the population either attaining or closely approaching the optimal solution.

### 3.2 Local exploitation

**Fig. 1:**
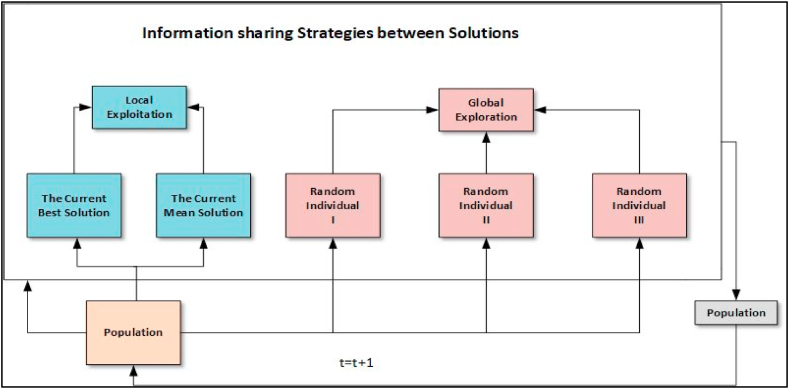
The framework of the proposed GNDO [35].

The proposed structure of GNDO is clearly presented in Fig. 1. As seen, GNDO upholds a simplistic design, with its information exchange mechanisms in local exploitation and global exploration particularly designed for GNDO's functionality. The foundation for local exploitation lies within the developed generalized normal distribution model, which is primarily guided by the current mean position and the existing optimal position. Conversely, global exploration is associated with three individuals selected at random. A comprehensive breakdown of these two learning strategies is presented below: Local exploitation embodies the pursuit of finding improved solutions within the search area that contains all current individual positions. The creation of a generalized normal distribution model for optimization is based on the relationship between the distribution of individuals within the population and the normal distribution (Eq. (3)).

(3) $$v_{i}^{t}=\mu_{i}+\delta_{i}\times \eta ,i=1,2,3,\ldots ,N$$

In this context, $v_{i}^{t}$ denotes the trail vector of the ith individual at a given time t, $\mu_{i}$ is the generalized mean position of the ith individual, $\delta_{i}$ symbolizes the generalized standard variance, and η is the penalty factor. Moreover, $\mu_{i}$ , $\delta_{i}$, and η can be defined as Eqs. (4), (5) and (6) respectively:

(4) $$\mu_{i}=\frac{1}{3}\left(x_{i}^{t}+x_{\text{Best}}^{t}+M\right)$$

(5) $$\delta_{i}=\sqrt{\frac{1}{3}\left[{\left(x_{i}^{t}-\mu \right)}^{2}+{\left(x_{\text{Best}}^{t}-\mu \right)}^{2}+{\left(M-\mu \right)}^{2}\right]}$$

(6) $$\eta =\left\{ \begin{array}{c} \sqrt{-\log\left(\lambda_{1}\right)}\times \cos\left(2\pi \lambda_{2}\right),\text{if}\;a<=b\\ \sqrt{-\log\left(\lambda_{1}\right)}\times \cos\left(2\pi \lambda_{2}+\pi \right),\text{otherwise} \end{array}\right.$$

where a, b, $\lambda_{1}$ and $\lambda_{2}$ are random numbers within the range of 0 and 1, $x_{\text{Best}}^{t}$ is the current best location, and M denotes the average location of the current population. Furthermore, the calculation of the mean location, M, can be carried out with the following procedure as Eq. (7),

(7) $$M=\frac{\sum_{i=1}^{N}\;x_{i}^{t}}{N}$$

### 3.3 Global exploration

In the context of GNDO, a random approach is employed to achieve equilibrium between exploration and exploitation. The search space is globally scrutinized to identify promising areas. Global exploration within GNDO is dependent on three randomly selected individuals (Eq. (8)).

(8) $$v_{i}^{t}=x_{i}^{t}+\underset{\text{Local}\;\text{information}\;\text{sharing}}{\underbrace{\beta \times \left(\left|\lambda_{3}\right|\times v_{1}\right)}}+\underset{\text{Global}\;\text{information}\;\text{sharing}}{\underbrace{\left(1-\beta \right)\times \left(\left|\lambda_{4}\right|\times v_{2}\right)}}$$

where $\lambda_{3}$ and $\lambda_{4}$ are two random numbers derived from a standard normal distribution, β (known as the adjust limit) is a random number between 0 and 1, and $v_{1}$ and $v_{2}$ are two trail vectors. The determination of trail vectors, $v_{1}$ and $v_{2}$, is done as per Eqs. (9) and (10)

(9) $$v_{1}=\left\{ \begin{array}{c} x_{i}^{t}-x_{p1}^{t},\text{if}f\left(x_{i}^{t}\right)<f\left(x_{p1}^{t}\right)\\ x_{p1}^{t}-x_{i}^{t},\text{otherwise} \end{array}\right.$$

(10) $$v_{2}=\left\{ \begin{array}{c} x_{p2}^{t}-x_{p3}^{t},\text{if}f\left(x_{p2}^{t}\right)<f\left(x_{p3}^{t}\right)\\ x_{p3}^{t}-x_{p2}^{t},\text{otherwise} \end{array}\right.$$

where p1, p2, and p3 are three unique random integers from 1 to N, adhering to the condition p1≠p2≠p3≠i.The tradeoff between the exploration and exploitation in GNDO is achieved randomly.

### 3.4 Multi-objective generalized normal distribution optimization (MOGNDO)

**Fig. 2:**
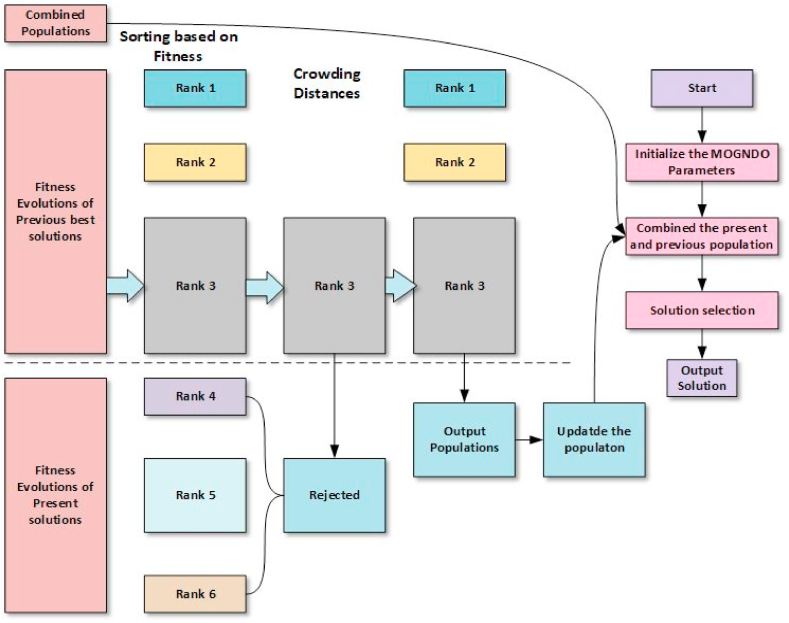
The procedure of non-dominated sorting approach.

The Multi-Objective Generalized Normal Distribution Optimization (MOGNDO) represents an innovative approach in optimization techniques, utilizing the strengths of the elitist non-dominated sorting (NDS) strategy and the crowding distance (CD) mechanism. MOGNDO is engineered to manage multiple concurrent objectives that might be in conflict, which makes it an extremely adaptable instrument in an array of intricate problem-solving instances. The cornerstone of MOGNDO is the broad application of the normal distribution model, a fundamental principle in statistical analysis and probability theory. This model provides a way to understand and interpret the positions of individuals within a search space and refines these positions according to a series of established objectives. The use of the NDS strategy is a crucial aspect of MOGNDO. This elitist technique is utilized for tackling multi-objective optimization issues. The operation of the NDS strategy revolves around the ranking of solutions based on a principle of dominance. According to this principle, a solution is deemed superior if it outperforms another in at least one objective without trailing behind in any other. This method of ranking effectively sieves through potential solutions, eventually selecting a collection of ‘non-dominated' or ‘Pareto-optimal' solutions. Conversely, the Crowding Distance (CD) mechanism is instrumental in preserving the diversity amongst the chosen solutions. This mechanism determines the density of solutions encircling a specific solution within the objective space, which ensures a broad dispersion of solutions throughout the Pareto front. It thereby avoids the congregation of solutions and guarantees a thorough portrayal of the trade-offs amongst objectives. Ultimately, the implementation of MOGNDO based on the NDS strategy and CD mechanism presents a robust and efficient method for addressing multi-objective optimization problems. It strikes a balance between identifying the most optimal solutions (exploitation) and venturing into uncharted territories in the search space (exploration), thus offering a variety of high-quality solutions. The definition of the CD mechanism is given as per Eq. (11).

(11) $$CD_{j}^{i}=\frac{{fobj}_{j}^{i+1}-{fobj}_{j}^{i-1}}{{fobj}_{j}^{\max}-{fobj}_{j}^{\min}}$$

where ${fobj}_{j}^{\max}$ and ${fobj}_{j}^{\min}$ are the maximum and minimum values of $j^{\text{th}}$ objective function. Fig. 2 provides a visual interpretation of a method underpinned by Non-Dominated Sorting (NDS).

**Fig. 3:**
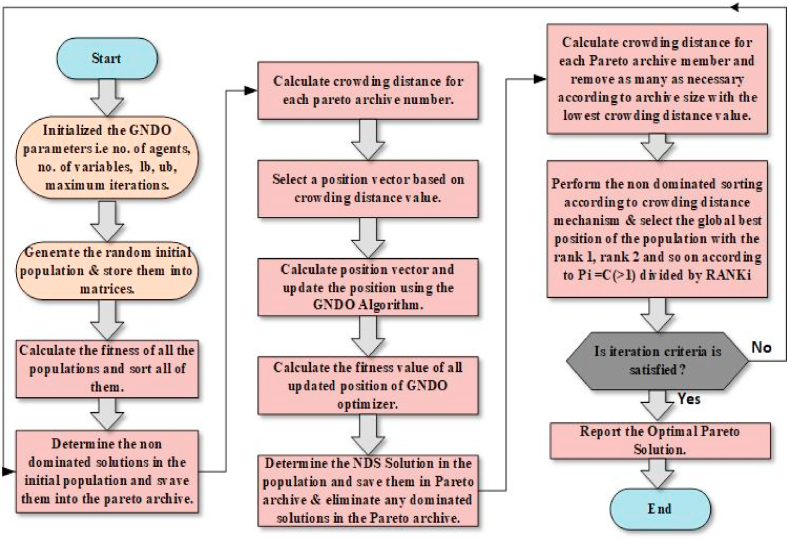
Flowchart of MOGNDO algorithm.

Algorithm-II details the pseudocode for executing the MOGNDO algorithm. The initiation of the MOGNDO methodology includes determining the requisite inputs, such as the population size (Np), the parameters for termination, and the upper limit for the number of iterations or generations (Maxit). Subsequently, each objective function within the objective space vector F for the parent population Po is assessed, using a randomly generated Po within the feasible search space region S. The next phase involves subjecting Po to the elitist-inspired Crowding Distance (CD) and NDS. This is followed by the amalgamation of Po with a newly formed population Pj, yielding population Pi. Pi is then sorted, using the data derived from CD, NDS, and elitist non-dominance. The top Np options are evaluated to formulate a new parent population. This cycle continues until the termination conditions are met. A comprehensive illustration of the MOGNDO process can be found in Fig. 3.

**Table:** 

Algorithm-II: Pseudocode of MOGNDO
Step 1: Initially Generate population ($P_{0})$ randomly in solution space (S) Step 2: Evaluate objective space (F) for the generated population (Po) Step 3: Sort the based on the elitist non-dominated sort method and find the non-dominated rank (NDR) and fronts Step 4: Compute crowding distance (CD) for each front Step 5: Update solutions $\left(P_{j}\right)$ Step 6: Merge $P_{0}$ and $P_{j}$ to create $P_{i}=P_{0}\cup P_{j}$ Step 7: For $P_{i}$ perform step 2 Step 8: Based on NDR and CD sort $P_{i}$ Step 9: Replace $P_{0}$ with $P_{i}$ for $N_{p}$ first members of $P_{i}$

### 3.5 Constraint handling approach

A substantial number of engineering design challenges in real-world scenarios are multi-objective and have considerably nonlinear constraints. Successfully resolving such constrained Multi-Objective Problems (MOPs) necessitates the management of all constraints within their defined parameters. Although there are diverse constraint handling methodologies outlined in the available literature, the MOGNDO algorithm implements a static penalty technique. This method transforms a constrained issue into an unconstrained one by imposing a significant penalty on the pertinent objective function whenever a constraint is breached. Here is a detailed explanation of the static penalty method as Eq. (12):

(12) $$f_{j}\left(X\right)=f_{j}\left(X\right)+\sum_{i=1}^{p}\;P_{i}\;\max\left\{g_{i}\left(X\right),0\right\}+\sum_{i=p}^{NC}\;P_{i}\;\max\left\{\left|h_{i}\left(X\right)\right|-\delta ,0\right\}$$

where $f_{j}\left(X\right),j=1,2\ldots n$ stands for the objective function set for optimization (with a focus on minimization here), $X=\left\{x_{1},x_{2},\ldots x_{m}\right\}$ symbolizes the design variables, $g_{i}\left(X\right)⩽0,i=1,2\ldots p$ indicates the inequality constraints, $h_{i}\left(X\right)=0,i=p+1\ldots NC$ refers to the equality constraints, and ***δ*** signifies the allowable deviation in the equality constraint.

## 4 Results and discussion

The computational simulations for the comparison of GNDO with other state of the art are executed on a Personal Computer equipped with 4 GB of RAM and a processor operating at 3.20 GHz. The algorithms under investigation, including MOGNDO, ALO, IMO, SCA, and their multi-objective versions, were implemented using MATLAB R2020b.

### 4.1 MOGNDO results for test benchmark problems

MOGNDO method is initially applied to the benchmark unconstrained test function as detailed in Ref. [36] to gauge its efficacy before tackling real-world problems. To validate the efficiency and accuracy of the MOGNDO method, seven benchmark unconstrained test functions (namely DZDT1, DZDT2, DZDT3, DZDT4, DZDT5, DZDT6, and DZDT7) are utilized, as they are deemed to be representative standards in this context. The solution to the optimization problem lies in the adept adjustment of the parameter values that govern the algorithm. Consequently, we have undertaken a comparative study of various scenarios, maintaining the size of each population as a constant. After careful analysis, we have concluded that a population size of 40 and a maximum iteration count of 100 are the most effective for our unconstrained test benchmark functions. The archive size is considered as 30. To solve the benchmark functions, each unrestricted test is subjected to 10 trials. It is worth mentioning here that mean solution of the current population and the current optimal solution are employed by GNDO to generate the next generation population. In addition, GNDO, do not need any effort for fine tuning initial parameters. Adjust limit (β) plays a significant role in determining the balance between local and global exploration. A value closer to 1 would emphasize local information, while a value closer to 0 would prioritize global information. We found that there exists an optimal range for each parameter that ensures a good balance between exploration and exploitation, leading to both fast convergence and accurate solutions.

To ascertain the degree of convergence accomplished by the MOGNDO algorithm, several performance metrics are taken into consideration. These include Generational Distance (GD), Inversion Generational Distance (IGD), Spacing Metrics (SP), Diversity Metrics (DM), and Spread Metrics (SD). Both GD and IGD are pivotal for determining the closeness of the obtained Pareto solutions to the true Pareto front. While GD measures the average distance from the obtained solutions to the nearest points on the true Pareto front, IGD quantifies the average distance from the points on the true Pareto front to the nearest obtained solutions. These metrics help in quantifying the quality and extent of convergence of the solutions and are essential for ensuring that the solutions we obtain are not just optimal but also as close as possible to the ideal solutions. SP measures the spread of solutions in the Pareto front. A well-distributed set of solutions is desirable because it provides decision-makers with diverse options, allowing for better flexibility and adaptability in making informed decisions. It also ensures that our algorithm doesn't get stuck at a local optimum but explores the entire solution space. DM and SD metrics gauge the diversity and spread of the solutions across the Pareto front. Achieving diverse solutions is critical in multi-objective optimization as it ensures that the optimization process does not overly favor one objective over another, but rather seeks a balance, ensuring solutions are spread uniformly across the Pareto front.

**Fig. 4:**
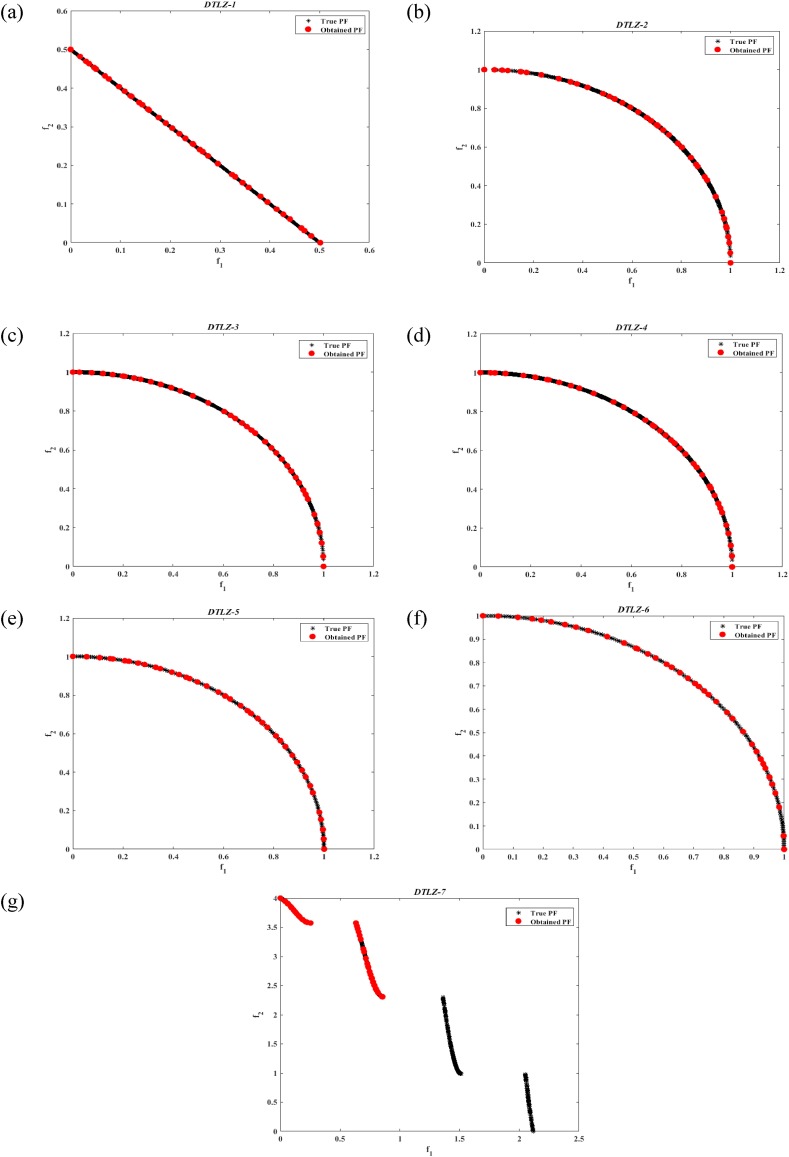
Pareto optimal front obtained by MOGNDO algorithm for various Test functions (a) DTL-Z1 (b) DTL-Z2 (c) DTL-Z3 (d) DTL-Z4 (e) DTL-Z5 (f) DTL-Z6 (g) DTLZ-7.

The data showcased in Table 1, Table 2, Table 3, Table 4, Table 5 suggest that the MOGNDO algorithm consistently achieves outstanding results in terms of performance measures, such as convergence and solution correctness, including metrics like GD and IGD, SP, DM, and SD. Therefore, the MOGNDO method can guarantee optimal convergence across all test functions. Fig. 4 present the outcomes (archive solutions) for all seven benchmark test problems. These visuals demonstrate that the MOGNDO approach is capable of approximating the Pareto front, which is a measure of optimal solutions. Moreover, a comparison of Pareto front estimations substantiates that the MOGNDO method can deliver commendable performance. Hence, the MOGNDO method exhibits significant potential for practical applications, particularly in complex engineering challenges such as wheel design problems associated with BLDC motors. It can be concluded that the MOGNDO method holds the upper hand in terms of efficiency, effectiveness, and adaptability in such practical applications.

**Table 1:** Results of GD metric on test functions (DTL-Z1 to DTLZ-7).

Test Functions	Minimum	Average	Median	Maximum	Std Dev
DTLZ-1	0.00015484	0.00017462	0.00017462	0.00019439	2.7967e−05
DTLZ-2	0.00022877	0.00035435	0.00035435	0.00047994	0.0001776
DTLZ-3	0.00013201	0.0001599	0.0001599	0.00018778	3.9439e−05
DTLZ-4	0.00013363	0.00017287	0.00017287	0.00021211	5.5497e−05
DTLZ-5	0.00041784	0.00041935	0.00041935	0.00042087	2.1367e−06
DTLZ-6	0.00041753	0.00043149	0.00043149	0.00044544	1.9732e−05
DTLZ-7	6.8505e−05	7.8393e−05	7.8393e−05	8.8282e−05	1.3984e−05

**Table 2:** Results of IGD metric on test functions (DTL-Z1 to DTLZ-7).

Test Functions	Minimum	Average	Median	Maximum	Std Dev
DTLZ-1	0.00043414	0.00045295	0.00045295	0.00047176	2.6606e−05
DTLZ-2	0.00040735	0.0004279	0.0004279	0.00044844	2.9062e−05
DTLZ-3	0.00043936	0.00045733	0.00045733	0.0004753	2.5418e−05
DTLZ-4	0.00047169	0.00048034	0.00048034	0.00048899	1.2231e−05
DTLZ-5	0.0010278	0.001031	0.001031	0.0010342	4.4958e−06
DTLZ-6	0.0010654	0.001125	0.001125	0.0011846	8.4288e−05
DTLZ-7	0.0089284	0.0089448	0.0089448	0.0089612	2.3198e−05

**Table 3:** Results of spacing metric on test functions (DTL-Z1 to DTLZ-7).

Test Functions	Minimum	Average	Median	Maximum	Std Dev
DTLZ-1	0.00046326	0.00082096	0.00082096	0.0011787	0.00050587
DTLZ-2	0.10574	0.11699	0.11699	0.12825	0.015918
DTLZ-3	0.12152	0.12559	0.12559	0.12967	0.0057624
DTLZ-4	0.1043	0.11776	0.11776	0.13121	0.019027
DTLZ-5	0.14492	0.14558	0.14558	0.14623	0.00092568
DTLZ-6	0.091708	0.099164	0.099164	0.10662	0.010544
DTLZ-7	0.37909	0.38372	0.38372	0.38836	0.006555

**Table 4:** Results of diversity metric on test functions (DTL-Z1 to DTLZ-7).

Test Functions	Minimum	Average	Median	Maximum	Std Dev
DTLZ-1	0.38266	0.39428	0.39428	0.4059	0.016429
DTLZ-2	0.34012	0.37209	0.37209	0.40405	0.045203
DTLZ-3	0.31235	0.33655	0.33655	0.36075	0.034221
DTLZ-4	0.34308	0.37275	0.37275	0.40242	0.041963
DTLZ-5	0.29606	0.32274	0.32274	0.34942	0.037731
DTLZ-6	0.37796	0.38725	0.38725	0.39655	0.013142
DTLZ-7	0.76784	0.78969	0.78969	0.81155	0.030904

**Table 5:** Results of spread metric on test functions (DTL-Z1 to DTLZ-7).

Test Functions	Minimum	Average	Median	Maximum	Std Dev
DTLZ-1	0.37154	0.38525	0.38525	0.39895	0.019384
DTLZ-2	0.33354	0.36407	0.36407	0.39459	0.043166
DTLZ-3	0.30492	0.32993	0.32993	0.35493	0.035368
DTLZ-4	0.33543	0.36229	0.36229	0.38916	0.037991
DTLZ-5	0.29065	0.31645	0.31645	0.34224	0.036482
DTLZ-6	0.36879	0.37914	0.37914	0.3895	0.014641
DTLZ-7	0.79726	0.81795	0.81795	0.83865	0.029267

### 4.2 MOGNDO algorithm results for brushless motor wheel design problem

Following the analysis of various test cases, the design optimization of the Brushless Direct Current (BLDC) motor is carried out employing four distinct multi-objective algorithms, including the proposed Multi-Objective Generalized Normal Distribution Optimization (MOGNDO) method. Each of the selected algorithms is executed over a series of 10 iterations, with the governing parameters for all algorithms being configured in alignment with the specifications laid out in Table 6. Given that the design problem of the BLDC wheel motor comprises six constraints, a constraint management mechanism is integrated with the proposed methodology. In this research endeavor, the fuzzy membership technique is paired with the proposed algorithm in order to derive the Best Compromise (BC) outcomes. Once the Pareto solutions are obtained, and the BC is located among the non-dominated solutions, which are subject to change during the decision-making stage, these are leveraged to establish BC outcomes across the trade-off features. The subsequent section delves into the results derived from the application of the proposed algorithm and the other selected algorithms to the design problem of the BLDC wheel motor. Some of the algorithms selected for practical application include Generalized Normal Distribution Optimization (GNDO), Ant Lion Optimizer (ALO) [37], Ion Motion Optimization (IMO) [38], and Sine Cosine Algorithm (SCA) [39]. However, these algorithms have yet to be assessed in the context of BLDC wheel motor design challenges. Each of the selected algorithms is promptly put into action to solve the design problem at hand and to optimize the design variables, such as minimizing the overall mass of the motor or maximizing its efficiency. The objective functions and control parameters are subjected to 10 iterations of the respective algorithms, as tabulated in Table 6. The selection of the optimal control parameters is informed by multiple test runs and literature reviews. All of the algorithms that emerged victorious managed to adeptly navigate challenging engineering problems. To extend the evaluation of the efficiency of all algorithms, the BLDC motor design described in next section. All algorithms are executed ten times for each of the two target problems, as previously mentioned.

**Table 6:** Performance analysis of mono-objective (Efficiency maximization) BLDC wheel motor design problem.

Control Variables	GNDO	ALO	IMO	SCA
$B_{d}$ (T)	1.800	1.717	1.799	1.800
$B_{e}$ (T)	0.648	0.644	0.649	0.721
$B_{cs}$ (T)	0.899	1.460	1.003	1.225
$D_{s}$ (mm)	0.201	0.207	0.203	0.197
ζ (A/mm^2^)	2042333.325	2000000.000	2024299.648	2183685.962
Efficiency in % (η)	**95.318**	95.235	95.312	95.185

#### 4.2.1Case 1 : maximizing the motor efficiency

In this particular case study, the design variables of the Brushless Direct Current (BLDC) motor are optimized by incorporating the objective function 1 as depicted in Eq. (1). Algorithms selected for this task, namely the GNDO, ALO [37], IMO [38] and SCA [39] are directly applied to objective function 1. The optimized design variables resulting from each of the 10 individual runs are chronicled in Table 6. The results that demonstrate the optimal performance from the 10 runs are indicated in bold in each table. As evident from Table 6, the most notable motor efficiency is garnered by the GNDO, ALO [37], IMO [38] and SCA [39] algorithms, yielding efficiencies of 95.318%, 95.235%, 95.312%, and 95.185%, respectively. From the results, it becomes clear that the GNDO algorithm delivers superior efficiency in the design of the BLDC motor wheel when compared to the other tested algorithms. This conclusion is drawn from a comprehensive analysis and comparative assessment of the efficiency rates yielded by the respective algorithms.

#### 4.2.2Case 2 : minimizing the motor mass

**Fig. 5:**
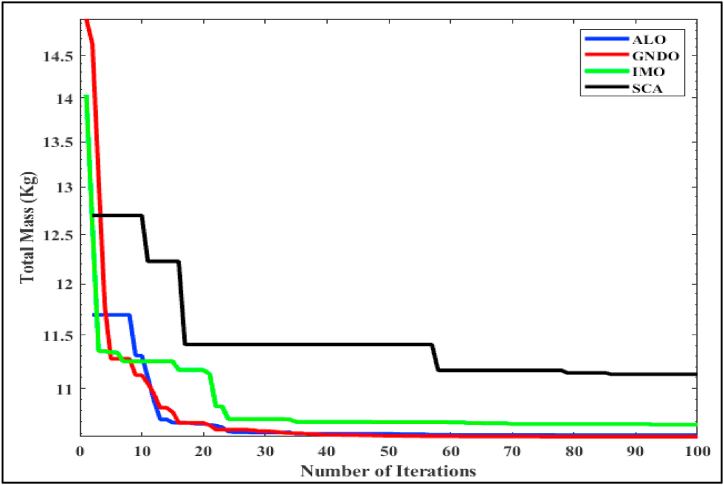
Convergence characteristics of mass minimization.

In this illustrative study, we focused on the optimization of design variables for Brushless Direct Current (BLDC) motors. We specifically paid attention to Objective Function 2, as described in Equation (2). Our methodology paralleled our approach in the previous case study, where we directly applied an assortment of algorithms - namely the GNDO, ALO [37], IMO [38], and SCA [39] - to Objective Function 2. The application of these algorithms across ten independent runs yielded a set of optimized design variables, as detailed in Table 7. The standout results from each table are highlighted in bold for clarity. Table 7, in particular, reveals a notable finding - the lowest motor mass (10.569 kg) was achieved using the GNDO algorithm when its iteration count was at 10. On the other hand, the ALO, IMO, and SCA algorithms produced slightly higher motor masses of 10.584 kg, 10.677 kg, and 11.132 kg, respectively. This comparison of outcomes brings the effectiveness of the GNDO algorithm to light, suggesting it is superior for this specific objective. Fig. 5 offers additional insight into this phenomenon by showcasing the convergence curve for all selected algorithms. From this illustration, it's evident that the GNDO algorithm reaches the point of minimal mass more swiftly compared to the ALO, IMO, and SCA algorithms. Based on these findings, it can be inferred that the GNDO algorithm demonstrates superior performance in tackling Objective Function 2 for the BLDC motor design optimization problem.

**Table 7:** Performance analysis of mono-objective (total mass minimization) BLDC wheel motor design problem.

Control Variables	GNDO	ALO	IMO	SCA
$B_{d}$ (T)	1.800	1.800	1.794	1.800
$B_{e}$ (T)	0.652	0.649	0.647	0.656
$B_{cs}$ (T)	1.600	1.599	1.595	1.144
$D_{s}$ (mm)	0.189	0.193	0.198	0.185
ζ (A/mm^2^)	3797344.536	3989267.473	4299175.092	3473617.170
Total mass in Kg ($M_{tot}$)	**10.569**	10.584	10.677	11.132

#### 4.2.3Case 3 : multi-objectives optimization for minimizing the motor mass and maximization of efficiency

**Fig. 6:**
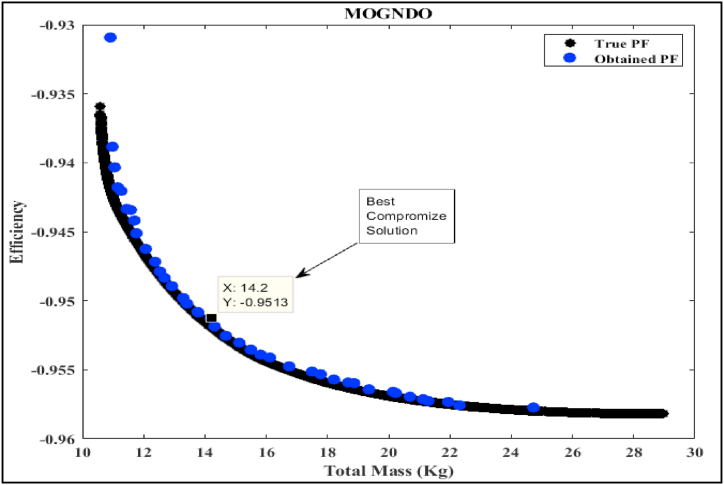
Pareto optimal front obtained by MOGNDO algorithm.

**Fig. 7:**
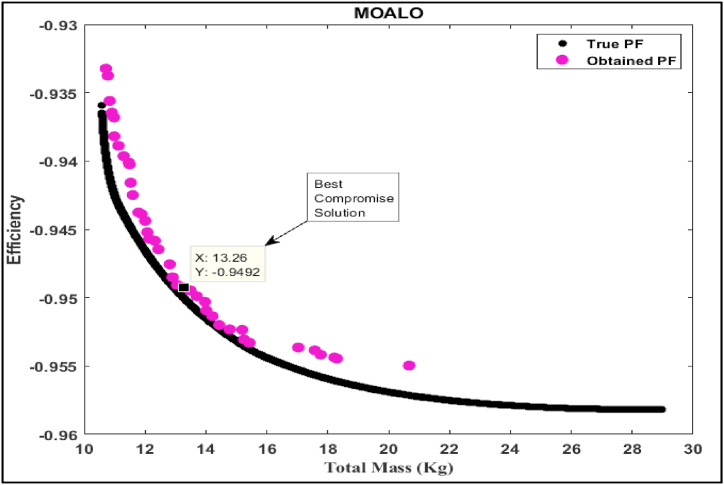
Pareto optimal front obtained by MOALO algorithm.

**Fig. 8:**
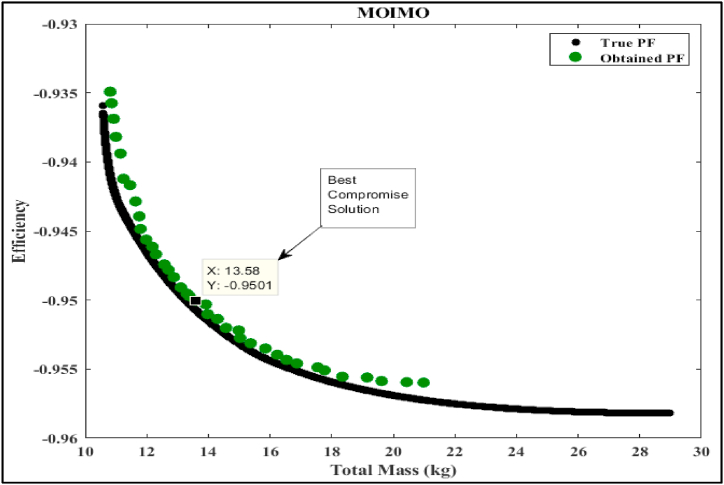
Pareto optimal front obtained by MOIMO algorithm.

**Fig. 9:**
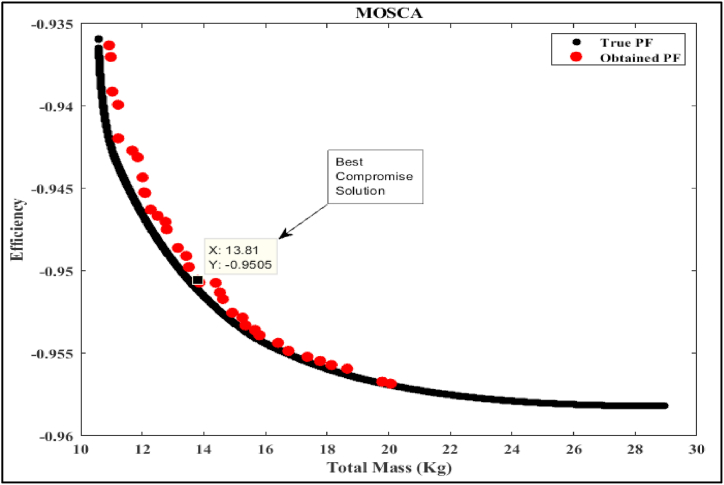
Pareto optimal front obtained by MOSCA algorithm.

In this part of the study, we delve into the simulation analysis of the proposed Multi-Objective Generalized Normal Distribution Optimization (MOGNDO) algorithm. We also provide a comparative analysis of its performance against other competitive algorithms, such as Multi-Objective Ant Lion Optimizer (MOALO), Multi-Objective Ion Motion Optimization (MOIMO), and Multi-Objective Sine Cosine Algorithm (MOSCA). Retaining the parameters consistent with the mono-objective optimization problem (i.e., an archive size of 100, a maximum iteration count of 100, and a population of 30 individuals), we conducted ten runs of the multi-objective optimization problem. The optimal compromise solutions from these runs are outlined in Table 8, which also provides insight into the most plausible alternatives. This table also includes values of the objective functions – namely motor efficiency, motor mass, and the balance between motor mass and efficiency – as well as the five design/optimization variables ($B_{e}$, $D_{s}$, $B_{d}$, $B_{cs}$, and ζ) that were optimized using all four algorithms. Upon examining the results, it is clear that the proposed MOGNDO algorithm surpasses its counterparts in performance. Consequently, we can deduce that MOGNDO offers a more satisfactory resolution to the design complexities of the BLDC motor. Fig. 6, Fig. 7, Fig. 8, Fig. 9 display the Pareto fronts for all the algorithms, which are vital for obtaining the best Pareto solutions. To realize these solutions, which are represented in the objective space shown in Fig. 6, Fig. 7, Fig. 8, Fig. 9, the decision variables require optimization. The proposed algorithm incorporates a static penalty scheme, demonstrating an effective mechanism for managing constraints. Progress appears consistent with the proposed algorithm, showing promising strides towards optimizing both motor efficiency and mass. In conclusion, these findings substantiate the superior performance of the proposed MOGNDO algorithm in contrast to the other algorithms under consideration.

**Table 8:** Performance analysis of multi-objective (Efficiency maximization and total mass minimization) BLDC wheel motor design problem.

Control Variables	MOGNDO	MOALO	MOIMO	MOSCA
$B_{d}$ (T)	1.792	1.800	1.784	1.735
$B_{e}$ (T)	0.671	0.667	0.674	0.657
$B_{cs}$ (T)	1.017	1.011	0.958	1.404
$D_{s}$ (mm)	0.200	0.193	0.193	0.198
ζ (A/mm^2^)	2359767.730	2568380.927	2497227.844	2269842.591
Efficiency in % (η)	**95.125**	94.924	95.006	95.054
Total mass in Kg ($M_{tot}$)	14.198	**13.263**	13.582	13.808

Table 9 shows the results of Wilcoxon Signed-Ranks Test (WSRT) between the efficiency values and motor mass values obtained using our proposed MOGNDO method and other algorithms (MOALO, MOIMO, MOSCA) over the 10 runs. The p-values obtained from the WSRT-tests were consistently below the 0.05 threshold, indicating a statistically significant difference between the results produced by MOGNDO and other algorithms, it is concluded that MOGNDO can show a competitive and robust performance when compared with MOALO, MOIMO, and MOSCA.

**Table 9:** **S**tatistical validation of MOGNDO using Wilcoxon signed-ranks test.

MOGNDO vs.	*R*^+^	*R*^−^	*p*-value
MOALO	151	105	0.445
MOIMO	182	68	0.058
MOSCA	131	119	0.907

### 4.3 Computational complexity of GNDO

The computational complexity is a key metric for GNDO algorithm to evaluate its run time and position update, which is based on the number of individuals N, variables D, and maximum number of iterations $T_{\max}$. In each iteration, N individuals need to update their positions and N comparisons are performed. Thus, the total computation complexity of GNDO can be expressed as $O\left(NDT_{\max}+NT_{\max}\right)$ and MOGNDO algorithm for M objectives is O(M*N*N). The space complexity of GNDO is the maximum space measure used by any one algorithm directive during iteration, so the total space complexity of GNDO is O(ND).

## 5 Conclusions and future works

Based on the extensive work carried out in this study, the main contributions can be summarized as:

• A meticulous comparative analysis of multiple optimization algorithms in the context of BLDC motor design, with GNDO emerging as the most effective.
• Introduction and successful application of the MOGNDO method, demonstrating its robustness and adaptability in complex engineering challenges.
• A clear demonstration that MOGNDO can effectively balance between objectives, making it a promising tool for real-world BLDC motor design.
• Provision of comprehensive statistical metrics and visual aids (like Pareto fronts) to provide a holistic view of the performance of the tested algorithms.

This work can be further extended to explore the following avenues:

• A compelling avenue for future research would be to integrate the optimization results with modern control systems, aiming for even higher efficiency and performance metrics.
• The methods and findings could be scaled to industrial applications, where BLDC motors of varying scales and sizes are deployed, to ascertain the generalizability of our conclusions.
• Utilizing machine learning models to predict performance based on design parameters might augment the optimization process, making it faster and more intuitive.

A key limitation of this study is that despite MOGNDO algorithm's superiority in our testing environment, it may involve higher computational complexity due to its multi-objective nature. This might affect its real-time applicability in scenarios where rapid optimization is critical. Our research primarily focused on the BLDC motor design problem with specific constraints and objectives. It's yet to be determined how well MOGNDO scales to larger, more complex systems with additional constraints and objectives.

Nevertheless, this work has significant implications for real-world applications. The MOGNDO algorithm, given its capability to simultaneously manage multiple objectives, provides an optimal balance between operational efficiency and mass of the BLDC motor. This ensures that the motor designed using this algorithm will not only perform efficiently but will also be lightweight, potentially leading to energy savings and increased longevity. The study's findings highlight the practicality and applicability of MOGNDO, setting a new benchmark for efficiency and optimization in the motor design industry.The MOGNDO source code is available at: https://github.com/kanak02/MOGNDO.

## Funding

European Union under the REFRESH – Research Excellence For REgion Sustainability and High-tech Industries project number via the Operational Program Just Transition.: CZ.10.03.01/00/22_003/0000048.

## Institutional review board statement

Not applicable.

## Informed consent statement

Not applicable.

## Data availability statement

The data presented in this study are available through email upon request to the corresponding author.

## CRediT authorship contribution statement

**Sundaram B. Pandya:** Conceptualization, Formal analysis, Investigation, Methodology, Writing – original draft. **Pradeep Jangir:** Conceptualization, Methodology, Software, Writing – review & editing. **Miroslav Mahdal:** Conceptualization, Methodology, Software, Writing – review & editing. **Kanak Kalita:** Software, Writing – review & editing. **Jasgurpreet Singh Chohan:** Formal analysis, Investigation, Software, Writing – original draft, Writing – review & editing. **Laith Abualigah:** Software, Writing – review & editing.

## Declaration of competing interest

The authors declare that they have no known competing financial interests or personal relationships that could have appeared to influence the work reported in this paper.
